# Innovative Multi-Site Photoplethysmography Analysis for Quantifying Pulse Amplitude and Timing Variability Characteristics in Peripheral Arterial Disease

**DOI:** 10.3390/diseases6030081

**Published:** 2018-09-17

**Authors:** Michael Bentham, Gerard Stansby, John Allen

**Affiliations:** 1 Faculty of Medical Sciences, Newcastle University, Newcastle upon Tyne NE2 4HH, UK; m.bentham@ncl.ac.uk; 2 Northern Vascular Centre, Freeman Hospital, Newcastle upon Tyne NE7 7DN, UK; gerard.stansby@nuth.nhs.uk; 3 Northern Medical Physics and Clinical Engineering, Freeman Hospital, Newcastle upon Tyne Hospitals NHS Foundation Trust, Newcastle upon Tyne NE7 7DN, UK

**Keywords:** cardiovascular variability, heart-rate variability, peripheral arterial disease, photoplethysmography, pulse

## Abstract

Photoplethysmography (PPG) is a simple-to-perform vascular optics measurement technique that can detect blood volume changes in the microvascular bed of tissue. Beat-to-beat analysis of the PPG waveform enables the study of the variability of pulse features, such as the amplitude and the pulse arrival time (PAT), and when quantified in the time and frequency domains, has considerable potential to shed light on perfusion changes associated with peripheral arterial disease (PAD). In this pilot study, innovative multi-site bilateral finger and toe PPG recordings from 43 healthy control subjects and 31 PAD subjects were compared (recordings each at least five minutes, collected in a warm temperature-controlled room). Beat-to-beat normalized amplitude variability and PAT variability were then quantified in the time-domain using two simple statistical measures and in the frequency-domain bilaterally using magnitude squared coherence (MSC). Significantly reduced normalized amplitude variability (healthy control 0.0384 (interquartile range 0.0217–0.0744) vs. PAD 0.0160 (0.0080–0.0338) (*p* < 0.0001)) and significantly increased PAT variability (healthy control 0.0063 (0.0052–0.0086) vs. PAD 0.0093 (0.0078–0.0144) (*p* < 0.0001)) was demonstrated for the toe site in PAD using the time-domain analysis. Frequency-domain analysis demonstrated significantly lower MSC values across a range of frequency bands for PAD patients. These changes suggest a loss of right-to-left body side coherence and cardiovascular control in PAD. This study has also demonstrated the feasibility of using these measurement and analysis methods in studies investigating multi-site PPG variability for a wide range of cardiac and vascular patient groups.

## 1. Introduction

Photoplethysmography (PPG) is a simple-to-perform vascular optical measurement technique that is often used to detect blood volume changes in the microvascular bed [1]. PPG can use a low-cost light-emitting diode (LED) and photodiode configuration to measure the light absorption and transmission in tissue, with changes in light detected by the PPG photodiode to provide a pulse waveform with features that can be extracted and utilized diagnostically [2]. Although the PPG signal is not fully understood, it has been used by many researchers to provide useful information about the cardiovascular system [1]. PPG can be performed using a single site measurement or using multiple measurement sites from across the body, for example, the right and left ear lobes, index finger pads, and great toe pads [1].

The PPG waveform has a pulsatile ‘alternating current (AC)’ component superimposed on a low-frequency ‘direct current (DC)’ component [2]. Beat-to-beat analysis of the AC waveform produces a number of features that have been described in the literature, for example, timing, amplitude, and shape, and the variability of each of these. Amplitude, typically measured from the foot to the systolic peak of the pulse, is an indicator of arterial blood flow and tissue volume changes [3]. Studies have found that amplitude can be used as a measure in blood pressure or stroke volume estimation and also as an indicator of sympathetic tone [3,4]. Another important characteristic of the PPG waveform is the pulse arrival time (PAT), which has been measured using the foot or the peak of the pulse. PAT requires an ECG as well as a pulse and is often measured from the ECG R-wave peak [5]. A short PAT has been shown to link well with increased blood pressure [6] and has also been shown to relate to arterial stiffness and compliance [7,8,9]. Allen et al. [10] found PAT increased with disease severity and was linked to a reduced arterial compliance and BP reductions due to arterial stenosis. Pulse width and pulse area can also both be calculated from the PPG waveform. Studies have shown that both the pulse width and pulse area increase when the peripheral vascular resistance changes [11,12]. The variability of PPG waveforms is body site specific [13] and studying the signals from a range of bilateral body sites simultaneously is of considerable interest in photoplethysmography research.

The more slowly varying constituents of the PPG signal, often group together and called the DC component, have also been studied in detail, including in the frequency-domain, for example, using wavelet and/or conventional Fourier Transform based analyses, that have been reported for heart rate variability (HRV) and laser Doppler flowmetry (LDF) studies. Key specific frequency band ranges for LDF were first described in 1999 [14] and have since been redesigned with the discovery of oscillations at very low frequencies (the suggested different bands and their attributed physiological origin are summarized in Table 1). The frequency band ranges can differ between individuals and be modified by exercise and some disease processes, therefore, an ideal universal division of the frequency ranges is impossible [15]. However, this type of quantification using the frequency domain provides a promising way forward to identify physiological processes and particularly those at the lower frequencies of cardiovascular variability. Nitzan et al. has published key research in the analysis of the low frequency and beat-to-beat changes in PPG signals from different peripheral body sites, and his work has included the assessment of such oscillations in healthy subjects and in diabetic patients. Nitzan and co-workers studied baseline and amplitude fluctuations from PPG signals and demonstrated that these low frequency (0.02–0.05 Hz) oscillations were mediated by sympathetic activity [16,17], with studies before and after sympathectomy supporting this [18]. Nitzan also demonstrated that the cross-correlation of pulse signal changes between right-left body sides was high in healthy individuals, but this reduced significantly in diabetic patients [19]. There is considerable further scope to utilize time and/or frequency domain pulse wave analysis techniques to study a wide range of cardiovascular related diseases.

Peripheral arterial disease (PAD) is a common condition that can severely affect the quality of life and also life expectancy. PAD affects as many as 17.3 per 10,000 person years in the UK [20], although incidence is thought to be as high as 15–20% in people over 70 years of age, with just a quarter of them displaying symptoms [21,22]. PAD is associated with several atherosclerotic co-morbidities, including coronary artery disease and cerebrovascular disease, with a prevalence of 40% to 60% in PAD patients [21]. The first diagnostic test performed for PAD is often the Ankle Brachial Pressure Index (ABPI). ABPI is the ratio of ankle BP to brachial BP, and a value of less than 0.9 is indicative of PAD [23]. ABPI is a non-invasive, inexpensive method that can have as high as 95% sensitivity and 99% specificity, when compared to computed tomography angiography (CTA), but is not without its limitations [23,24]. ABPI is user-dependent, therefore, it is prone to potential error, and is non-diagnostic in patients with heavily calcified vessels because the arteries are non-compressible [24]. Imaging is considered the gold-standard diagnostic test, but is often only used in patients being considered for surgery [25]. Duplex ultrasound is a safe and effective test often used for surveillance, but can be highly operator-dependent; therefore, CTA or magnetic resonance angiography (MRA) are often used to confirm and localize disease for surgical planning [26]. However, both CTA and MRA are expensive, time consuming, and invasive [27]. CTA is particularly limited due to the use of iodinated contrast, which is nephrotoxic and can cause acute kidney injury [27]. PPG technology is potentially offering new diagnostic tools for the low-cost, non-invasive, and safe assessments of PAD, with the capability to be accessible and portable. One way that PPG is being used is as an automated, user-independent method of measuring BP for ABPI calculations in PAD, demonstrating that PPG is at least as effective as Doppler ultrasound and can be more effective than manual measurements [28,29,30]. More recently, pulse wave analysis (PWA) has been used as a tool for diagnosing PAD, with changes in pulse amplitude, PAT, shape, and a number of other pulse features having been shown to relate to the presence of atherosclerosis, meaning that PPG is a useful non-invasive diagnostic tool [31,32,33,34,35].

This exploratory study aims to quantify and contrast the cardiovascular variability of multi-site PPG waveform features in healthy subjects and patients with peripheral arterial disease. Variability will be assessed in the time domain and also in the frequency domain for a unique dataset of multisite PPG recordings collected from previous PPG studies by Dr John Allen’s Newcastle-based Vascular Optics research group.

## 2. Materials and Methods

### 2.1. Participants and Datasets

This exploratory pilot study used a unique dataset comprising of data collected from various former multi-site PPG studies by the Newcastle Vascular Optics group. The subject recruitment has previously been detailed by Nath et al. [34] and Allen et al. [35]. In summary, patients were recruited from the Newcastle upon Tyne Hospitals NHS Foundation Trust and healthy participants recruited from hospital staff groups, the University of the Third Age (U3A, Wearside Branch), and the Institution of Engineering and Technology (IET) retired members group. Ethical approvals had been obtained for the earlier data collection studies and each participant gave their written informed consent. The multi-site PPG pulse recordings, extracted resampled data, and clinical data sets are not currently available outside of the Vascular Optics group.

### 2.2. Multi-Site PPG System and Measurements

**Figure 1:**
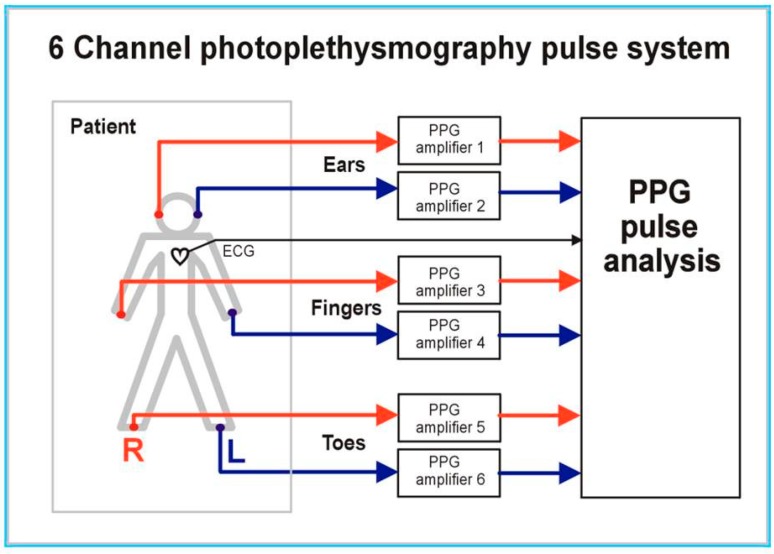
Multi-site photoplethysmography (PPG) pulse measurement and analysis system (from [1], with permission from IoP Publishing).

**Figure 2:**
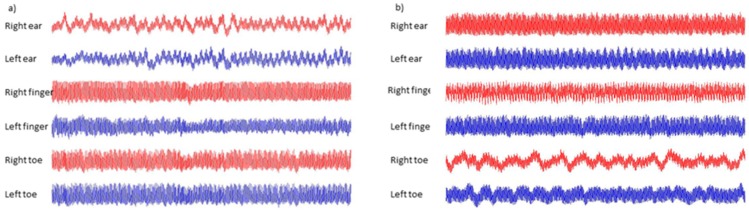
An example of the original multi-site PPG recordings over a period of five minutes for (**a**) a healthy control and (**b**) a peripheral arterial disease (PAD) patient. Note: The amplitude levels for each body site in (**a**,**b**) are scaled to fit the plot and do not represent the gain normalized levels.

The innovative multi-site PPG measurement system set-up and measurements have also been previously described in the literature by Nath et al. [34] and Allen et al. [35], and were originally developed by Allen and Murray [13]. The multi-site PPG system employed was a six-channel measurement system, facilitating the recording of pulse waveforms from the tissue pads of bilateral ear lobes, index fingers, and halluces (see Figure 1 and Figure 2). The PPG probes were in reflection mode and with a near infrared (950 nm) LED operating wavelength. The PPG amplifier bandwidth was 0.15 to 20 Hz, with pulse channels matched electronically and optically for the 3 pairs of right/left side channels so that differences between body sides were likely to be physiological rather than due to systematic bias from the equipment.

The recordings were all made with the patient in the supine position, in a warm temperature (25 ± 1 °C) controlled room with a minimum 10 min acclimatization period. The recordings were made for at least 5 min to a computer using 16-bit analogue-to-digital conversion at either a 2000 or 2500 Hz sampling rate, depending on which former study was used to collect the data. A single lead ECG was measured simultaneously to provide a cardiac timing reference for the subsequent pulse wave analysis (PWA). The gain of the pulse amplifiers was also recorded and used in the analysis.

### 2.3. Pulse Wave Analysis and Signal Quality Control

**Figure 3:**
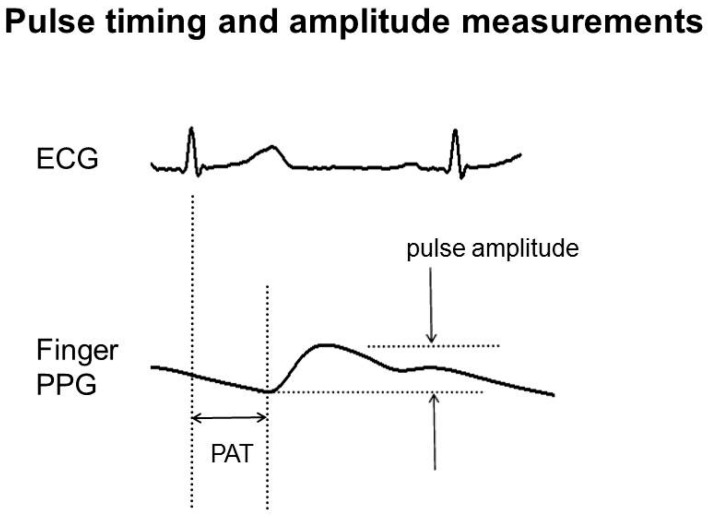
Example PPG pulse arrival time (PAT) and amplitude measurements for a single pulse from the finger site. Amplitude is normalized by dividing by the associated amplifier gain setting. Timing and normalized amplitude measurements are subsequently resampled at 4 Hz for the time and the frequency domain analyses.

Pulse wave analysis was carried out exclusively using bespoke research software (MATLAB, 2012a version, MathWorks Inc., Natick, MA, USA). Prior to the beat-to-beat feature extraction of the pulse timing and amplitude data (see Figure 3), a semi-automatic process of quality control was performed to minimize noise related artifact, and to identify poor datasets to be excluded from the final analysis [10]. This QA checking process was performed on all recordings prior to the time domain and frequency domain analyses, where occasional noisy beats were interpolated out. Beat-to-beat amplitude and timing measurement quality can be affected by factors, such as excessive movement during recording, cardiac arrhythmias (such as atrial fibrillation or ventricular ectopic beats), and, occasionally for severe vascular disease, when a pulse cannot be detected easily (‘a flat liner’).

Our group focused specifically on index finger and great toe recordings rather than ear pad recordings because PAD typically presents in the lower limbs, and occasionally upper limbs, of patients [36]. In addition, artifact/noise appeared to affect the ear site significantly more than other sites, the morphologies at the ears appeared more complicated than more distal sites, the ear site links only to the external carotid artery rather than the internal carotid artery, and it was considered that they would have added little clarity to this pilot study evaluation. Furthermore, previous literature also assessing the use of PPG for PAD diagnosis have more commonly used finger and toe sites compared to ear sites [31,32,33,34,35].

Quality control was an extensive and meticulous process that involved manually checking every pulse for the bilateral finger and toe site recordings using a bespoke MATLAB GUI-based processing program, previously written by Allen et al. [10], and has been used in numerous other published studies [13,35,37]. Toe site recordings were first quality checked and were excluded if they did not meet the acceptable quality suitable for further consideration in the study. Subjects whose toe signals passed the quality control stage then had their finger signals quality checked and further exclusions were made where necessary.

The multi-site PPG pulse wave analysis software is not currently available outside of the Vascular Optics group.

#### 2.3.1. Time-Domain Analysis

Beat-to-beat PAT over the full recording was calculated from the ECG R wave to the foot of each pulse. Beat-to-beat amplitude was calculated from the difference in height between the 1st dominant peak and the foot of each pulse; this amplitude was then normalized using the respective site PPG amplifier gain settings noted at the time of the reading, with this normalization allowing individuals to be fairly summarized within a group and compared between groups [35]. From each site, the median values for the beat-to-beat PAT and normalized amplitude measures were calculated. To quantify the variability of each measure, the simple statistical methods of the standard deviation (SD) and interquartile range (IQR) of the PAT and amplitude over the length of a pulse recording and were calculated. Both SD and IQR measures were compared between subject groups; the former parametric and the latter non-parametric for data that might not have a normal distribution.

#### 2.3.2. Frequency-Domain Analysis

The magnitude squared coherence (MSC) was calculated to quantify the coherence of 5 min bilateral toe and finger PPG recordings for the frequency band ranges defined in Table 1 (Signal processing script using bespoke MATLAB signal processing scripts). MSC gives a value between 0 and 1 [38] for which an MSC value closer to 1 indicates more coherence between two signals for a specified frequency band. The frequency ranges were determined using previous work from Stefanovska et al. [14,39,40]: Very low frequency (VLF 0.0095–0.021 Hz); low frequency (LF 0.021–0.052 Hz); medium frequency (MF 0.052–0.145 Hz); high frequency (HF 0.145–0.6 Hz); and alternating current (AC 0.6–2.0 Hz). The frequency-domain analysis was performed on resampled beat-to-beat amplitude and PAT data (4 Hz) [41]. Right and left great toes and right and left index fingers were each compared using MSC. Overall, i.e., median MSC, estimates were then calculated for each frequency band and each subject group compared.

### 2.4. Statistical Analysis

Statistical analysis was carried out using the Minitab statistical package software (version 17). Data was tested for normality using the Anderson-Darling test and all variables were found to be non-parametric; therefore, only non-parametric statistical analysis was carried out. Continuous variables are reported as medians with the interquartile range (Q1–Q3) values. The Mann-Whitney U test was carried out to test for statistical significance between groups. A *p*-value <0.05 was considered to be statistically significant.

## 3. Results

### 3.1. Subject Demographics

Multisite PPG pulse recordings from 84 participants were originally included in the study. Ten subjects were excluded because of impaired quality toe signal recordings, the most common cause being linked to cardiac arrhythmia (n = 5), as well as excessive movement artifact type noise (n = 3) and signal ‘flat lining’ (n = 2; likely severe disease cases). Of the remaining 74 subjects, a further four recordings were excluded because of impaired quality based on finger site PPG measurements; however, the good quality toe signals from these subjects were still used for summary analysis at that site. Therefore, a total number of 148 toe and 140 finger QA checked body sites were available for the main analysis.

Of the 74 participants used in the final analysis, 43 were healthy controls (ABPI ≥ 0.9) and 31 participants had significant PAD in at least one leg (ABPI < 0.9). In the healthy control group, there were 20 males and 10 diabetic patients, with a median age of 68 (55–77) years. In the study PAD group, there were 15 males and 9 diabetic patients, with a median age of 69 (64–75) years. There was no significant difference in the overall ages for the two groups. The pilot study demographics are summarized in Table 2.

### 3.2. Toe Site PPG Variability in the Time-Domain Comparing PAD to Healthy Controls 

Variability was measured both in terms of the SD and the IQR in the recording period to compare the measurements. Overall, in both the SD and IQR variability in the toes, there was significantly lower amplitude variability and significantly higher PAT variability in PAD compared to the healthy control group (see Table 3).

**Figure 4:**
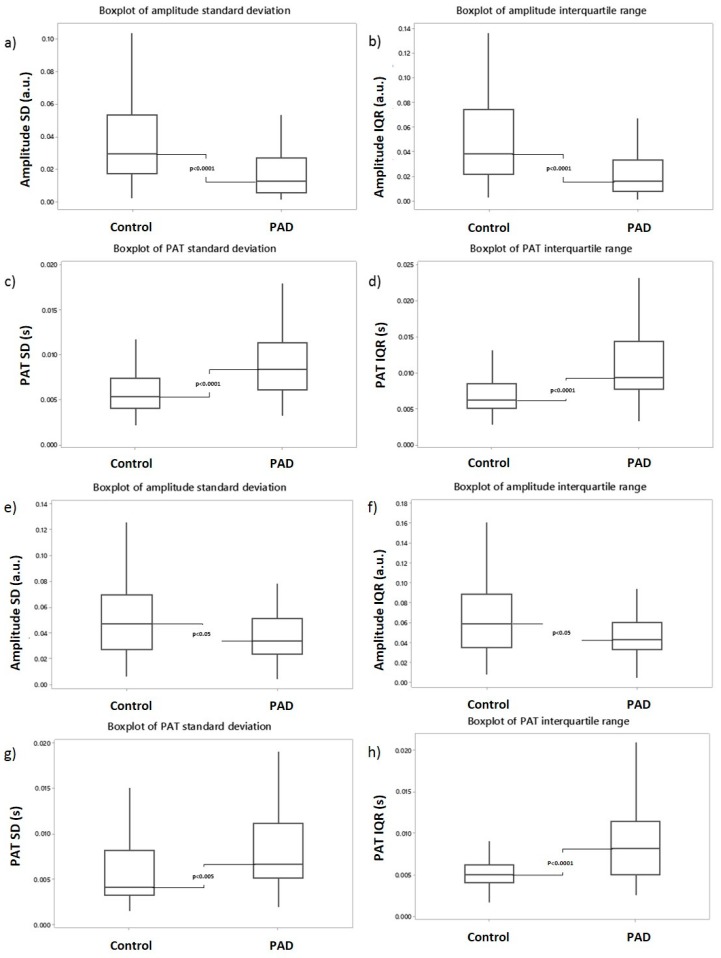
Boxplots of the variability for the PPG waveforms: (**a**) toe normalized amplitude variability (SD) was significantly lower in the PAD group compared to the healthy controls (*p* < 0.0001); (**b**) toe normalized amplitude variability (IQR) was also significantly lower in PAD (*p* < 0.0001); (**c**) toe PAT variability (SD) was significantly higher in the PAD group (*p* < 0.0001); (**d**) toe PAT variability (IQR) was also significantly higher in PAD (*p* < 0.0001); (**e**) finger amplitude (SD) was significantly lower in (**f**) finger amplitude (IQR) was significantly lower in PAD (*p* < 0.05); (**g**) finger PAT (SD) was significantly higher in PAD group (*p* < 0.005); and (**h**) finger PAT (IQR) was also significantly higher in PAD (*p* < 0.0001).

In this study, the normalized amplitude variability was significantly lower in PAD for both SD and IQR, with a median SD of 0.0124 (0.0056–0.0270) a.u. and a median IQR of 0.0160 (0.0080–0.0338) a.u. compared to the control group, which had median SD of 0.0295 (0.0172–0.0536) a.u. and a median IQR of 0.0384 (0.0217–0.0744) a.u. (see Figure 4). Statistical testing showed highly significant differences in both the SD and IQR, giving *p*-values of <0.0001. In contrast, the PAT variability was significantly higher in PAD for both the SD and IQR, with a median SD PAT of 0.0085 (0.0061–0.0114) s and a median IQR of 0.0093 (0.0078–0.0144) s compared to the significantly lower values in the control group of 0.0054 (0.0041–0.0074) s and 0.0063 (0.0052–0.0086) s for PAT SD and IQR, respectively. Again, both the PAT SD and IQR yielded highly significant *p*-values of <0.0001.

### 3.3. Changes in the Frequency-Domain with PAD for the Toe Site

The MSC was calculated using the resampled PAT and showed significantly lower overall values in the PAD group across the range of study frequencies. For resampled normalized amplitudes, these were found to be significantly lower for the VLF, LF and HF bands (see Table 4).

### 3.4. Finger Site PPG Variability in the Time-Domain Comparing PAD to Healthy Controls

Variability in the normalized amplitude and PAT for the fingers mirrors the results from the toe site analysis as summarized previously. There was also less overall variability in PAD for finger normalized amplitude, but with greater variability in the PAT (see Table 5).

Normalized amplitude variability, measured in both SD and IQR, was significantly lower in the PAD group. Median amplitude SD was 0.0336 (0.0239–0.0511) a.u. in the PAD group compared to 0.0473 (0.0275–0.0695) a.u. in the control group, and median amplitude IQR was 0.0435 (0.0331–0.0603) a.u. in the PAD group compared to 0.0591 (0.0353–0.0890) a.u. in the control group (see Figure 4). Both SD and IQR differences between the subject groups were statistically significant. There were also statistically significant differences in PAT variability between the control and PAD groups—similar to those found in the toe sites. PAT SD was higher in PAD patients, with a median of 0.0066 (0.0051–0.0111) s, compared 0.0041 (0.0033–0.0082) s in the healthy controls. PAT IQR was also increased in the PAD group, with a median value of 0.0082 (0.0050–0.0114) s compared to 0.0050 (0.0041–0.0062) s. The differences between the two groups for PAT SD and IQR measures were highly statistically significant (*p*-values at least to <0.005).

### 3.5. Changes in the Frequency-Domain with PAD for the Fingers

MSC values were lower in the PAD group across the range of frequency bands for resampled normalized amplitudes; however, there were only statistically significant differences for LF and MF bands. In the LF range (Table 6), the median amplitude MSC was 0.834 (0.664–0.924) for the healthy control group compared to 0.680 (0.487–0.787) in PAD (*p* < 0.005). Also for the MF bands, the healthy control group had a median amplitude MSC of 0.673 (0.548–0.768) compared to 0.532 (0.404–0.694) for the PAD group (*p* < 0.01). MSC values were also lower in the PAD group for all frequency ranges for PAT measurements; however, there were only statistically significant differences for the MF and HF frequency bands as are summarized in Table 6 (each *p* < 0.05).

## 4. Discussion

This exploratory pilot study has quantified the variability in multi-site PPG pulse waveforms in both time and frequency domains for healthy controls and patients with peripheral arterial disease. Clear differences between subject groups have been shown.

### 4.1. Variability in Time-Domain Measures in Health and Peripheral Arterial Disease

Quantification of the variability in PPG pulse amplitude and timing measurements in PAD has not been extensively studied or discussed in the literature. Both our SD and IQR measures of variability produced similar results in terms of demonstrating differences in variability in health and vascular disease. Pulse feature variability is considered to be derived from an autonomic function [16,17]—it was therefore expected that PAD patients would have less variability in all time domain measurements. The variability in amplitude was expected to decrease in PAD and this has been demonstrated by using both SD and IQR approaches to its quantification. The difference between groups in amplitude variability appeared slightly more prominent for the toes compared to the fingers. Variability is likely to be reduced in PAD patients because of a number of pathophysiological mechanisms, particularly autonomic dysfunction, as well as reduced intrinsic myogenic activity and NO-dependent endothelial activity [14]. Further study is warranted using the methodologies described in this paper for those who have peripheral autonomic neuropathy.

**Figure 5:**
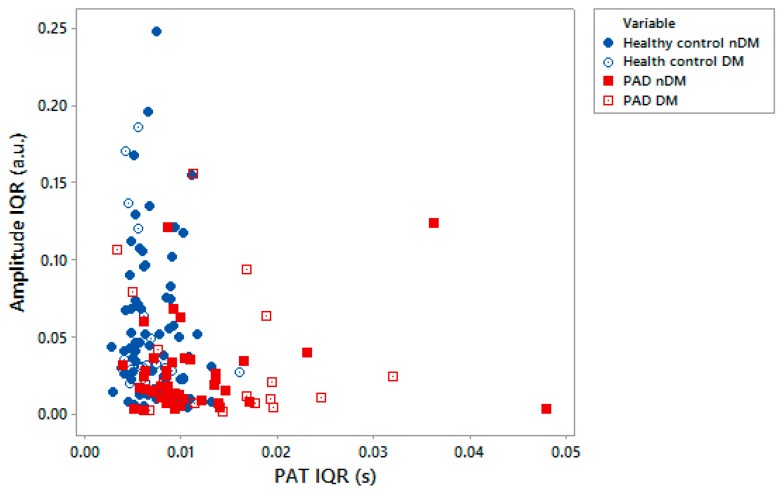
Scatterplot comparing amplitude IQR with PAD IQR demonstrating differences in PAT and normalized amplitude variability between healthy controls and PAD patients. Subjects with diabetes in the subject groups are represented as per the indicated legend symbols.

Interestingly, PAT variability was demonstrated to be higher overall for the PAD patient group compared to the healthy subjects. In both the toe and finger recordings, PAT variability was significantly higher in PAD patients at the toes and at the fingers. Timing variability is more difficult to explain as it was initially expected to decrease with the disease. This suggests that PAT variability in the time-domain may not be fully linked to sympathetic function and could also be affected by blood pressure changes over arterial stenosis/occlusion in PAD. Pulse profile landmark identification could also be a contributory factor since rounder pulse shapes can be seen in vascular patients [1]. We will aim to explore these observations further in a wider study of the analysis techniques employed. Comparing amplitude variability with PAT variability also helps demonstrate the distinction between the control and PAD patient groups (see Figure 5). The scatterplot shows the different cluster ranges, although with a degree of overlap, with lower PAT variability and higher normalized amplitude variability. In contrast, the PAD group has higher PAT variability and lower normalized amplitude variability. The reasons for these differences are not well understood and also warrant further study.

Although not the focus of this pilot study, we also assessed the effects on variability of systolic blood pressure (SBP) and diabetes mellitus (DM). SBP was found to be significantly higher in the PAD patient group, however, simple regression calculations found no clear correlations between this pressure and the time-domain variability measures. It was also found that, in the healthy control group, subjects with diabetes did not have significantly different time-domain variability compared to those without the condition.

### 4.2. Variability in the Frequency-Domain in Health and in PAD

The frequency analysis compared the MSC values from bilateral PPG measurements between the subject groups for each frequency band. Loss of coherence between bilateral sites (MSC tending towards 0) in a particular band suggests that the right-left body side synchronicity has been diminished, thus potentially indicating disease linked to the physiological origin for that frequency band range.

In the toes, normalized amplitude MSC values were significantly lower for the VLF, LF and HF frequency bands, while PAT MSC values were significantly lower across all frequency ranges for the PAD patient group. The finger site gave slightly different results to the toe site, with normalized amplitude MSCs being lower for LF and MF frequency bands in PAD, while PAT MSCs were lower for MF and HF bands in PAD. Lower amplitude MSC values found in the PAD patients suggest a loss of variability synchronicity for the bilateral finger and toe measurement sites. For the toes, the clearer differences between study groups was at the VLF and LF bands, which means amplitude variability could be attributed to NO-endothelial and sympathetic activity since both NO-endothelial function and sympathetic endothelial function are diminished in PAD. Finger MSC differences are at slightly higher frequency bands, which could suggest that the loss of NO-function could be less prominent in the upper limbs and a loss of sympathetic function and myogenic activity may also be factors here. 

### 4.3. Study Limitations

This study was a pilot to estimate the levels of PPG pulse variability, including testing if bilateral de-synchronization was present in peripheral arterial disease patients. Only a limited clinical demographic dataset was collected—for future research a larger cohort should be considered and to include a wider range of clinical variables to allow multivariate analyses to be explored with confidence. Similarly, a larger cohort of diabetic patients (with PAD and without PAD cases) should be studied to better establish if there are links to overall pulse variability. A few of the subjects in this pilot had notably high ABPI values, i.e., linked to rigid arteries, and future studies should study this aspect in appropriate detail to determine if PPG pulse variability is linked to arterial stiffness. In terms of the pulse wave analysis, we explored only a small number of basic variability measures—future research should consider a wider variety of techniques to further explore the differences between subject groups.

### 4.4. Future Directions of the Research

These derived datasets are very interesting and warrant further investigation as ultimately the toe pulse variability measures could add value to optimizing PAD diagnostics from simple PPG measurements, have utility in assessing response to therapy, and for enabling a better understanding of the pathophysiological mechanisms with the disease. Our proposed future direction is also to develop the pulse measurement and analysis technology using a wider cohort of patients for automated classifications in PAD, as well as for the automatic quantification of cardiovascular risk measures for innovative arterial stiffness, autonomic dysfunction, and endothelial dysfunction type assessments.

## 5. Summary

In this pilot study, the expected normal ranges for variability in multi-site PPG normalized pulse amplitude and PAT timing measures have been quantified, along with their expected ranges in patients with peripheral arterial disease. The observed de-synchronization in PAD of amplitude and timing variability between right and left body sides as assessed using MSC coherence frequency-domain methods has also been shown. These data suggest a complex picture of changes in vascular autoregulation, endothelial function and autonomic function in PAD, and highlights the compensatory mechanisms involved in trying to maintain adequate distal perfusion. The time-domain and frequency-domain PPG data determined in this pilot study should offer additional value for informing the design of future multi-site PPG studies in peripheral arterial disease, and for wider cardiovascular assessments.

## Figures and Tables

**Table 1:** Frequency intervals and their attributed physiological origin [14]. NO: Nitric oxide.

Frequency Band	Frequency (Hz)	Physiological Origin
**Alternating Current (AC)**	0.6–2.0	Heartbeat
**High frequency (HF)**	0.145–0.6	Respiratory activity
**Medium frequency (MF)**	0.052–0.145	Intrinsic myogenic activity
**Low frequency (LF)**	0.021–0.052	Neurogenic (sympathetic) activity
**Very low frequency (VLF)**	0.0095–0.021	NO-dependent endothelial activity

**Table 2:** Study participant demographics. NS: Non-significant.

Variable	Healthy Control (n = 43)	PAD (n = 31)	*p*-Value
**Age (years)**	68 (55–77)	69 (64–75)	NS
**Sex (male)**	20	15	NS
**Height (cm)**	169 (162–174)	167 (159–172)	NS
**Heart-rate (bpm)**	69 (64–77)	70 (61–79)	NS
**Systolic BP (mmHg)**	142 (124–158)	158 (144–177)	<0.001
**Minimum side ABPI**	1.07 (1.01–1.15)	0.59 (0.53–0.69)	<0.001

**Table 3:** Toe time-domain variability results comparing PAD and healthy control. Median (IQR) values are shown. a.u.: Arbitrary units.

Measure	Healthy Control Legs (n = 86)	PAD Legs (n = 62)	*p*-Value
**SD amplitude (a.u.)**	0.0295 (0.0172–0.0536)	0.0124 (0.0056–0.0270)	**<0.0001**
**IQR amplitude (a.u.)**	0.0384 (0.0217–0.0744)	0.0160 (0.0080–0.0338)	**<0.0001**
**SD PAT (s)**	0.0054 (0.0041–0.0074)	0.0085 (0.0061–0.0114)	**<0.0001**
**IQR PAT (s)**	0.0063 (0.0052–0.0086)	0.0093 (0.0078–0.0144)	**<0.0001**

**Table 4:** Magnitude squared coherence (MSC) analysis using resampled normalized amplitude and PAT frequency data for the toe site. Median (IQR) values are shown. NS: Non-significant.

Frequency Band	Healthy Control Legs (n = 86)	PAD Legs (n = 62)	*p*-Value
**Amplitude VLF**	0.685 (0.514–0.835)	0.466 (0.394–0.685)	**<0.005**
**Amplitude LF**	0.704 (0.563–0.799)	0.484 (0.342–0.704)	**<0.005**
**Amplitude MF**	0.611 (0.430–0.707)	0.536 (0.394–0.656)	NS
**Amplitude HF**	0.684 (0.545–0.839)	0.551 (0.444–0.735)	**<0.05**
**Amplitude AC**	0.526 (0.446–0.653)	0.497 (0.415–0.567)	NS
**PAT VLF**	0.870 (0.727–0.937)	0.701 (0.543–0.899)	**<0.05**
**PAT LF**	0.704 (0.546–0.882)	0.445 (0.269–0.646)	**<0.001**
**PAT MF**	0.631 (0.479–0.865)	0.435 (0.377–0.557)	**<0.001**
**PAT HF**	0.755 (0.575–0.904)	0.517 (0.468–0.699)	**<0.005**
**PAT AC**	0.685 (0.467–0.838)	0.426 (0.373–0.767)	**<0.01**

**Table 5:** Time-domain variability results in the fingers comparing PAD and healthy control groups. Median (IQR) values are shown. a.u.: Arbitrary units.

Measure	Healthy Control Arms (n = 78)	PAD Patient Arms (n = 62)	*p*-Value
**Amplitude SD (a.u.)**	0.0473 (0.0275–0.0695)	0.0336 (0.0239–0.0511)	**<0.05**
**Amplitude IQR (a.u.)**	0.0591 (0.0353–0.0890)	0.0435 (0.0331–0.0603)	**<0.0**5
**PAT SD (s)**	0.0041 (0.0033–0.0082)	0.0066 (0.0051–0.0111)	**<0.005**
**PAT IQR (s)**	0.0050 (0.0041–0.0062)	0.0082 (0.0050–0.0114)	**<0.0001**

**Table 6:** Resampled normalized amplitude and PAT frequency analysis for the finger site. Median (IQR) values are shown. NS: Non-significant.

Frequency Band	Healthy Control arms (n = 78)	PAD arms (n = 62)	*p*-Value
**Amplitude VLF**	0.804 (0.573–0.931)	0.696 (0.500–0.922)	NS
**Amplitude LF**	0.834 (0.664–0.924)	0.680 (0.487–0.787)	**<0.005**
**Amplitude MF**	0.673 (0.548–0.768)	0.532 (0.404–0.694)	**<0.01**
**Amplitude HF**	0.631 (0.515–0.782)	0.573 (0.458–0.699)	NS
**Amplitude AC**	0.492 (0.440–0.622)	0.486 (0.402–0.580)	NS
**PAT VLF**	0.750 (0.468–0.939)	0.633 (0.464–0.858)	NS
**PAT LF**	0.518 (0.325–0.746)	0.514 (0.380–0.637)	NS
**PAT MF**	0.581 (0.378–0.808)	0.439 (0.349–0.557)	**<0.05**
**PAT HF**	0.644 (0.499–0.833)	0.492 (0.398–0.733)	**<0.05**
**PAT AC**	0.540 (0.409–0.822)	0.434 (0.363–0.661)	NS
